# Merits and Demerits of ODE Modeling of Physicochemical Systems for Numerical Simulations

**DOI:** 10.3390/molecules27185860

**Published:** 2022-09-09

**Authors:** Kyuichi Yasui

**Affiliations:** National Institute of Advanced Industrial Science and Technology (AIST), Nagoya 463-8560, Japan; k.yasui@aist.go.jp

**Keywords:** modeling, ordinary differential equation (ODE), partial differential equation (PDE), numerical simulations, sonochemical reactions, mesocrystal, flexoelectric effect, ultrasound-assisted sintering, thermoacoustic engine

## Abstract

In comparison with the first-principles calculations mostly using partial differential equations (PDEs), numerical simulations with modeling by ordinary differential equations (ODEs) are sometimes superior in that they are computationally more economical and that important factors are more easily traced. However, a demerit of ODE modeling is the need of model validation through comparison with experimental data or results of the first-principles calculations. In the present review, examples of ODE modeling are reviewed such as sonochemical reactions inside a cavitation bubble, oriented attachment of nanocrystals, dynamic response of flexoelectric polarization, ultrasound-assisted sintering, and dynamics of a gas parcel in a thermoacoustic engine.

## 1. Introduction

One of the major developments in science and engineering in recent years is the development of numerical simulations using digital computers [1]. In association with the development of high-performance computers, first-principles calculations have been developed such as computational fluid dynamics (CFD), finite element method (FEM) applied to mechanics of materials, molecular dynamics simulations, density functional calculations for quantum mechanics, etc. [2,3,4,5,6,7,8,9,10,11,12,13,14,15,16,17,18,19,20,21,22,23,24,25,26,27]. Many of the fist-principles calculations are based on partial differential equations (PDEs). In addition, there are many other kinds of PDE modeling [28,29,30,31,32,33]. Conversely, modeling with ordinary differential equations (ODEs) has been reported in many fields of science such as biology, chemistry, physics, and material science [34,35,36,37,38,39,40,41,42,43,44,45,46,47,48,49,50,51,52,53,54,55,56,57,58,59,60,61,62,63,64,65,66,67,68,69,70,71,72,73,74,75,76,77,78,79,80,81,82,83,84,85,86,87,88,89,90,91,92,93]. In typical ODE modeling, the spatial uniformity of variables such as temperature and pressure is assumed, and the independent variable of the ODE systems is time in most applications [94]. The merits of ODE modeling compared to PDE modeling are as follows: it is computationally more economical, and it is much easier to trace important factors in numerical computations [80,81,95,96,97,98,99,100]. In other words, an ODE model is much more suitable for numerical simulations under various conditions compared to a PDE model [80,81,95]. In the present review, some examples of ODE modeling are discussed compared with PDE modeling in order to see the merits and demerits of ODE modeling. The selected topics are more or less hot ones. In Section 7, selected mathematical models are briefly described both for ODE and PDE models for comparison.

## 2. Chemical Reactions inside a Cavitation Bubble

**Figure 1:**
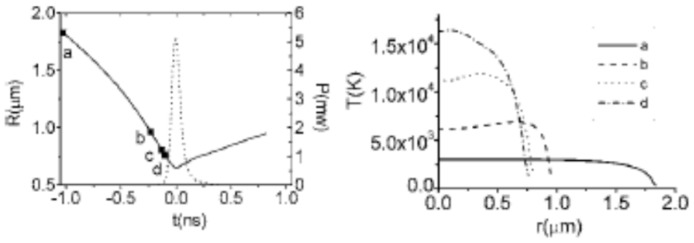
The results of numerical simulation of fundamental equations (PDE) for fluid mechanics inside a collapsing argon bubble under ultrasound (**left**) The bubble radius (solid line) and the optical pulse of sonoluminescence (dotted line). (**right**) The spatial profile of the calculated temperature at the given points of a, b, c, and d (filled squares in the left figure). Reprinted with permission from Ref. [109]. Copyright 2006, the American Physical Society.

**Figure 2:**
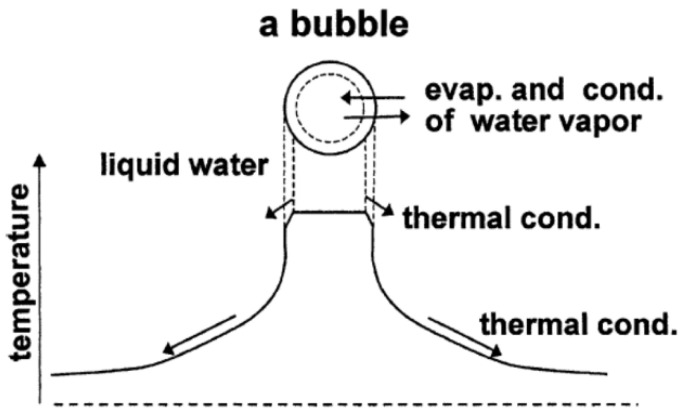
The model of bubble dynamics. Reprinted with permission from Ref. [75]. Copyright 2004, Elsevier.

The author has recognized the merits of ODE modeling through the research on chemical reactions inside a cavitation bubble [95]. The topic became popular soon after the experimental report on the extremely short pulse-width of single-bubble sonoluminescence (SBSL) by Barber and Putterman published in *Nature* in 1991 [101]. SBSL is the light emission phenomenon from a single stable bubble trapped near the pressure antinode of a standing ultrasonic wave, which was discovered by Gaitan and Crum in 1990 [102]. (Although the first report of SBSL was in 1962 by Yosioka and Omura [103], this work was not confirmed [104].) At the beginning of the SBSL research, the fundamental equations of fluid dynamics were numerically simulated inside a collapsing bubble, coupled with the Rayleigh–Plesset equation for the temporal evolution of bubble radius [105,106]. During the rarefaction phase of ultrasound, a bubble expands. At the compression phase of ultrasound, a bubble violently collapses. The reason for the violent collapse is the spherical geometry of a collapsing bubble and the inertia of the inflowing liquid [95]. According to the numerical simulations of the fundamental equations of fluid dynamics inside a collapsing bubble neglecting the effect of thermal conduction, a sharp spherical shock-wave is formed inside a bubble [105,106,107]. It converges at the bubble center, where temperature and pressure dramatically increase. It was proposed that the convergence and subsequent reflection of a spherical shock-wave is the reason for the extremely short pulse-width of SBSL. However, the shock-wave model resulted in considerably shorter pulse-width than the experimental data [105,106,107,108]. Furthermore, numerical simulations of the fundamental equations of fluid dynamics taking into account the effect of thermal conduction have shown that under many conditions of SBSL, a shock wave is absent inside a bubble because sound velocity increases as the distance from the bubble center decreases due to the increase in temperature caused by thermal conduction to the colder surrounding liquid [109,110]. As a pressure disturbance inwardly propagates with the local sound velocity plus the local fluid velocity from the bubble wall, it hardly overtakes previously radiated pressure disturbances, which prevents the formation of a sharp shock-wave inside a collapsing bubble [110]. An example of such a simulation is shown in Figure 1 [109]. It is seen that the temperature is nearly spatially uniform inside a collapsing bubble except at the thermal boundary layer near the bubble wall. In this case, the increase in bubble temperature is not by shock-wave convergence but mainly by pV work done by the inflowing liquid on a collapsing bubble [71]. With this in mind, an ODE model is practically useful, in which temperature and pressure are assumed to be spatially uniform inside a bubble except at the thermal boundary layer near the bubble wall (Figure 2) [71,72,73,74,75]. An ODE model is not applicable to the case when a shock-wave is formed inside a collapsing bubble. However, in the absence of a shock wave, an ODE model is computationally much more economical because spatial variation of temperature and pressure needs not to be simulated using a PDE model, which computationally costs due to its complexity. Furthermore, an ODE model is much more suitable to trace important factors compared to a PDE model. For example, the mechanism of heating of a bubble is much more easily traced. Under a condition of SBSL, a bubble is heated by pV work by 64%, by kinetic energy of inflowing liquid by 36%, and cooled by endothermic chemical reactions by 39%, by thermal conduction by 17% according to an ODE model, which are hardly traceable by a PDE model [71].

The demerit of ODE modeling is the accuracy of the model. ODE modeling versus PDE modeling is a trade-off between computational efficiency and physical accuracy. Thus, for ODE modeling, validation of the model is necessary by comparing with the experimental data. In the present case of chemical reactions inside a cavitation bubble, the experiment of single-bubble sonochemistry is suitable for the validation of the theoretical model. In 2002, Didenko and Suslick [111] experimentally reported in *Nature* that the amount of OH radicals produced from a SBSL bubble was $8.2\times 10^{5}$ per acoustic cycle in water at 3 °C irradiated by ultrasound of 52 kHz and 1.52 bar in frequency and pressure amplitude, respectively. The number of photons of SBSL light was $7.5\times 10^{4}$ per acoustic cycle [111]. In order to compare with the experimental data, numerical simulations were performed under the condition of the experiment using the ODE model [74]. In the ODE model, the following effects have been taken into account: thermal conduction both inside and outside a bubble, non-equilibrium evaporation and condensation of water vapor at the bubble wall, non-equilibrium chemical reactions inside a bubble, ionization of gases and vapor inside a bubble with considerable ionization-potential lowering, variation of liquid temperature at the bubble wall, and compressibility of liquid to the first order [71,72,73,74,75,112,113]. With regard to chemical reactions inside a bubble initially consisting of nitrogen, oxygen, argon, and water vapor, rates of 93 chemical reactions and their backward reactions were numerically calculated at each moment of the bubble collapse. The main bubble content inside a SBSL bubble is argon because nitrogen and oxygen chemically react inside a bubble and change to soluble species such as NOx and HNOx, which gradually dissolve into the surrounding liquid water. This argon rectification hypothesis has been validated both theoretically and experimentally [114]. Thus, the present numerical simulation was performed for an argon bubble with a tiny amount of nitrogen and oxygen [74].

**Figure 3:**
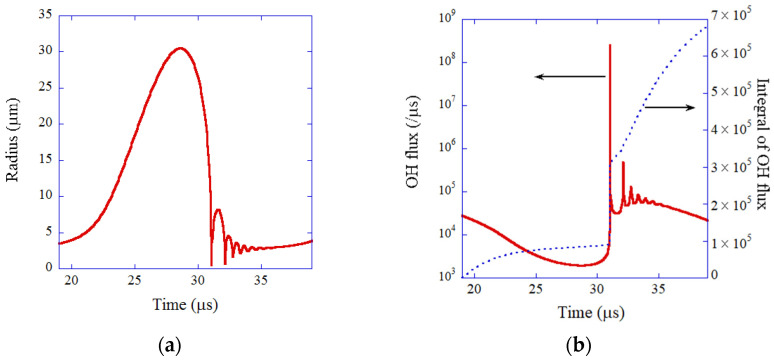
The results of numerical simulation of the ODE model for single-bubble sonochemistry for one acoustic cycle (**a**) The bubble radius. (**b**) OH flux and its time integral. Reprinted with permission from Ref. [74]. Copyright 2005, AIP Publishing.

**Figure 4:**
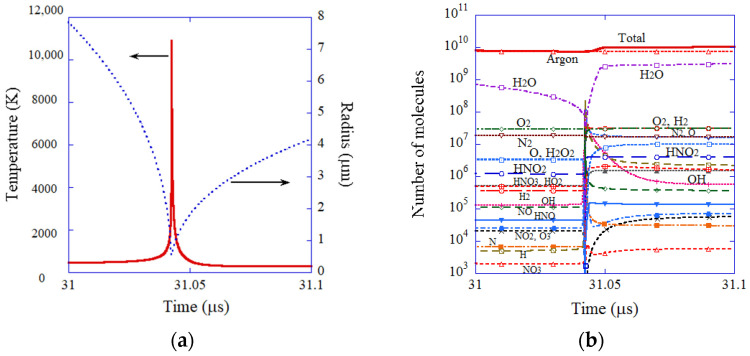
The results of numerical simulation of the ODE model for single-bubble sonochemistry at around the end of the violent bubble collapse (**a**) The bubble radius (blue dotted line) and temperature (red solid line). (**b**) The number of molecules inside a bubble with logarithmic vertical axis. Reprinted with permission from Ref. [74]. Copyright 2005, AIP Publishing.

The results of the numerical simulation are shown in Figure 3 and Figure 4 [74]. During the rarefaction phase of ultrasound, a SBSL bubble expands (Figure 3a). At the compression phase of ultrasound, a SBSL bubble violently collapses, which is followed by bouncing motion. The OH flux from the interior of a SBSL bubble to the surrounding liquid takes a sharp peak at the violent collapse (Figure 3b). About one-third of the total amount of OH radicals that diffuses into the surrounding liquid in one acoustic cycle diffuses out of a bubble at around the violent collapse. The other two-thirds diffuse out of a bubble during bubble expansion and bouncing motion. The total amount of OH radicals that diffuses into the surrounding liquid in one acoustic cycle is $6.6\times 10^{5}$ according to the present numerical simulation, which almost agrees with the experimental data $8.2\times 10^{5}$). Furthermore, the calculated number of photons is $8.0\times 10^{5}$, which also agrees with the experimental data ($7.5\times 10^{4}$). Thus, the present ODE model has been validated. It is beneficial for researchers because an ODE model is especially useful to scan the enormous parameter space of SBSL with moderate computational effort [80,81]. In Section 7.1 and Section 7.2, mathematical models are briefly described both for ODE and PDE models for comparison as well as the methods for the numerical solution.

## 3. Oriented Attachment of Nanocrystals

Mesocrystals are aggregates of nanocrystals in which crystal axes are aligned [115,116,117,118]. Mesocrystals have been intensively studied since 2005 [116,118]. One of the non-classical mechanisms for crystal growth is through the formation of mesocrystal and its subsequent fusion to a single crystal [118,119,120]. Thus, a firm understanding of the mechanisms for mesocrystal formation will be beneficial for many aspects of materials synthesis [119]. However, the mechanism by which nanocrystals are aligned with each other in mesocrystals remains controversial [115,116].

Self-assemblies are ordered structure of nanoparticles (nanocrystals) fabricated by dip-coating, drying-mediated self-assembly methods, Langmuir monolayer technique, etc. [116,121]. Mimura and Kato [122] fabricated self-assemblies of BaTiO_3_ nanocubes (nanocrystals) capped with oleic acid by dip-coating in organic solvent (mesitylene). The size of a BaTiO_3_ nanocube was about 15 nm, and the thickness of self-assemblies was 290 or 580 nm. After calcination at 400 °C for 1 h and sintering at 850 °C for 1 h in O_2_, the ordered structure of the self-assemblies was not changed except for the formation of the tight bonding between neighboring nanocubes [123]. The dielectric constant of the self-assemblies after the calcination and sintering was as high as 3800 and 2600 for 290 and 580 nm thick assemblies, respectively, at 1 MHz at room temperature [122]. The dielectric constant is much higher than the normal dielectric constant of a BaTiO_3_ bulk crystal (about 1600) without any domain contribution and those of typical BaTiO_3_ thin films (lower than 1000) [87]. It has been suggested that strain induced in each nanocube is the reason for the high dielectric constant, as discussed in the next section [86,87]. In the present section, the mechanism for the oriented attachment of BaTiO_3_ nanocubes in the self-assemblies is discussed based on numerical simulations using an ODE model [85].

The ODE model for the oriented attachment of BaTiO_3_ nanocubes capped with oleic acid in organic solvent (mesitylene) is as follows [85]. A collision of two nanocubes is considered. The equations of translational and rotational motion of a nanocube are given by the Newton’s equation of motion and the equation of rotational motion for a rigid body, respectively. In the Newton’s equation of motion, the gradients of the following interaction potentials are considered; electric dipole–dipole interaction between two colliding BaTiO_3_ nanocubes, van der Waals interaction, bridging interaction by oleic acid adsorbed on the surface of the nanocubes, steric repulsion due to oleic acid on the nanocube surface, and depletion force originated in the osmotic pressure due to large molecules (oleic acid) dispersed in the solution when large molecules are excluded from the narrow region between the two nanocubes. In addition, the random force causing the Brownian motion is considered. With regard to the equation of rotational motion for a rigid body, the following torques are considered: electric dipole–dipole interaction, van der Waals torque (Casimir torque), which originates in the anisotropy of dielectric constant and works to align the optical axes of two bodies, with the random torque causing the rotational Brownian motion. While the electric dipole–dipole interaction makes the two electric dipoles antiparallel, the van der Waals torque makes the two electric dipoles parallel or antiparallel because it solely makes the optical axes aligned [85,124,125,126].

**Figure 5:**
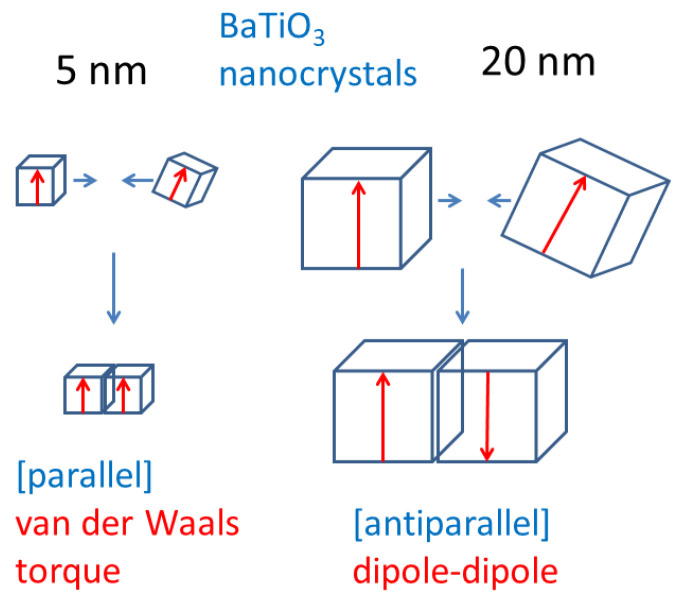
Summary of the results of numerical simulation of the ODE model for oriented attachment of BaTiO_3_ nanocrystals in organic solvent (mesitylene). Reprinted with permission from Ref. [85]. Copyright 2015, the American Chemical Society.

The results of the numerical simulations of the ODE model are summarized in Figure 5 [85]. When the size of a BaTiO_3_ nanocube is smaller than 5 nm, the crystal axes of two colliding BaTiO_3_ nanocubes are aligned in parallel or antiparallel by van der Waals torque. For larger size of BaTiO_3_ nanocubes, the electric dipoles of the two colliding nanocubes are aligned in antiparallel by electric dipole–dipole interaction. Thus, the self-assemblies of BaTiO_3_ nanocubes of 15 nm fabricated by dip-coating by Mimura and Kato [122] would be a kind of mesocrystal because the crystal axes are aligned.

**Figure 6:**
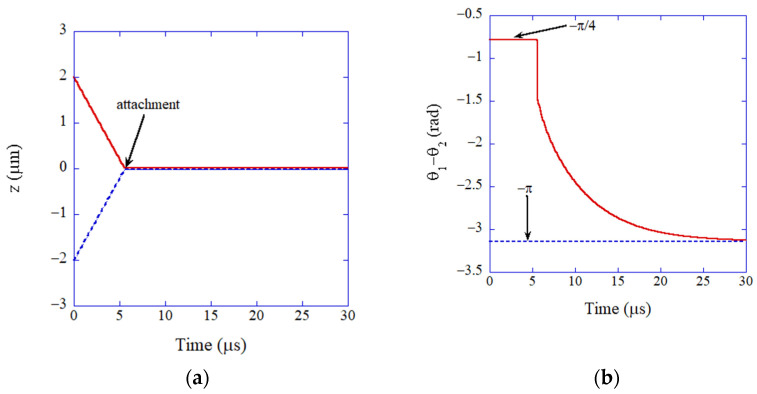

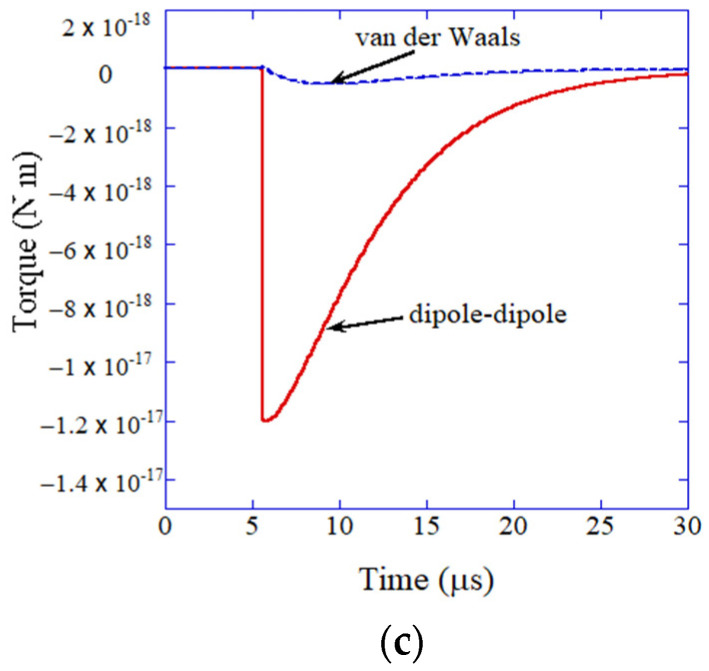
The results of numerical simulation of the ODE model for oriented attachment of BaTiO_3_ nanocrystals (20 nm) in organic solvent (mesitylene) as a function of time (**a**) The position of nanocrystals. (**b**) The relative angle of the electric dipoles. (**c**) The torque. Reprinted with permission from Ref. [85]. Copyright 2015, the American Chemical Society.

**Figure 7:**
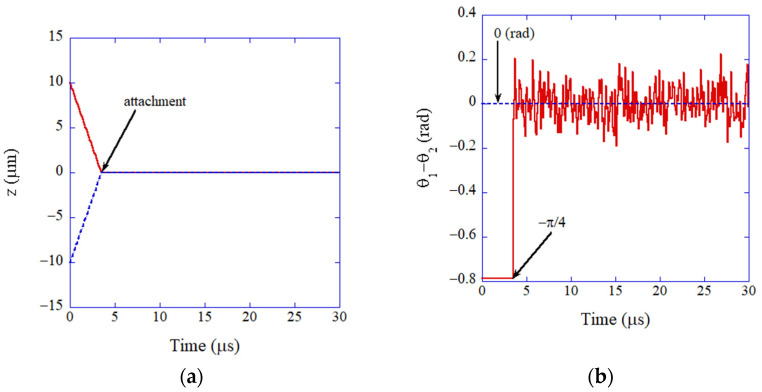
The results of numerical simulation of the ODE model for oriented attachment of BaTiO_3_ nanocrystals (5 nm) in organic solvent (mesitylene) as a function of time (**a**) The position of nanocrystals. (**b**) The relative angle of the electric dipoles. Reprinted with permission from Ref. [85]. Copyright 2015, the American Chemical Society.

Now, examples of the results of the numerical simulations of the ODE model are discussed (Figure 6 and Figure 7) [85]. The size of two colliding nanocubes is the same as 20 or 5 nm for Figure 6 and Figure 7, respectively. The electric dipole moment of a BaTiO_3_ nanocube for each size is calculated by the formula given in References [85,127]. The initial velocity of each nanocube is assumed as that for the Brownian motion [85].

For the case of 20 nm for the size of a nanocube (Figure 6), the two nanocubes attach at *t* = 5.6 μs in Figure 6a by the bridging force due to oleic acid on the surface of each nanocube [85]. After the attachment, the two electric dipoles are aligned in antiparallel (Figure 6b). It takes about 20 μs for the alignment (Figure 6b). The alignment is mostly due to electric dipole–dipole interaction (Figure 6c).

For the case of 5 nm (Figure 7), the two nanocubes attach at *t* = 3.5 μs in Figure 7a by the bridging force due to oleic acid as in the case of 20 nm. Contrary to the case of 20 nm, the two electric dipoles are aligned in parallel in much shorter time (less than 20 ns) (Figure 7b). The alignment is solely due to van der Waals torque because the electric dipole–dipole interaction only makes the two dipoles in antiparallel (After the attachment, some fluctuations are seen in Figure 7b because of the rotational Brownian motion). Thus, the summary in Figure 5 has been confirmed.

**Figure 8:**
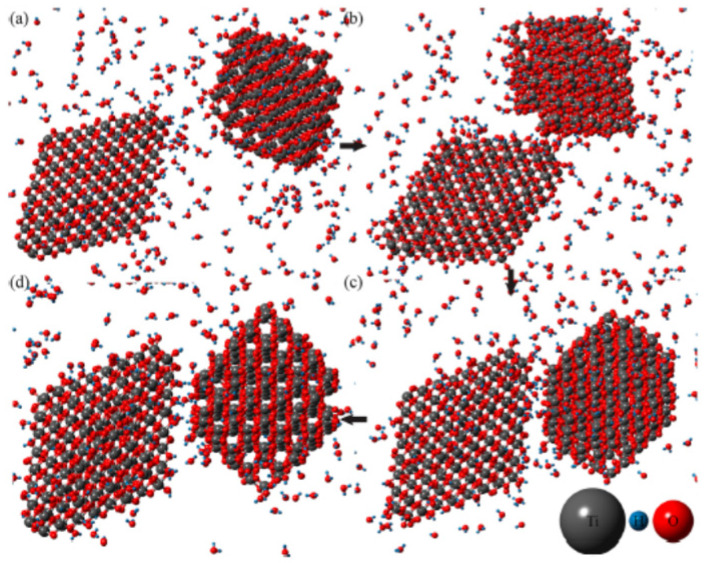
Snapshots of the results of molecular dynamics simulation for oriented attachment of TiO_2_ nanocrystals in the presence of water vapor These snapshots were taken at (**a**) 187.5 ps, (**b**) 205 ps, (**c**) 212.5 ps, and (**d**) 225 ps. Reprinted with permission from Ref. [128]. Copyright 2014, the American Chemical Society.

Next, the results of molecular dynamics simulations for oriented attachment of nanocrystals are briefly discussed in order to compare with those of the simpler ODE model. In molecular dynamics simulations, simulations of solvent molecules are practically difficult because of the computational complexity. Accordingly, instead of full simulations of solvent molecules, simulations in a humid environment with a much lower number of water molecules were performed as shown in Figure 8 [128]. Although the details of the oriented attachment could be studied by the molecular dynamics simulations, the assumed humid environment is different from the real solvent environment. Conversely, in the ODE model, the solvent environment is fully considered, and furthermore, it is computationally much more economical. As already seen in the previous section, an ODE model is much more suitable for numerical simulations under various conditions compared to the first principles calculations.

## 4. Dynamic Response of Flexoelectric Polarization

Flexoelectric polarization is polarization induced by strain gradient in dielectric crystals regardless of the crystal symmetry [129,130,131,132,133]. The magnitude of flexoelectric polarization is proportional to the strain gradient. The coefficient of the proportionality is called the flexoelectric coefficient. The flexoelectric coefficient for BaTiO_3_ has been experimentally reported as about 10 μC m^−1^ at room temperature [132]. Flexoelectric polarization may be important in micro/nano systems because strain gradient is considerably larger [129,130].

**Figure 9:**
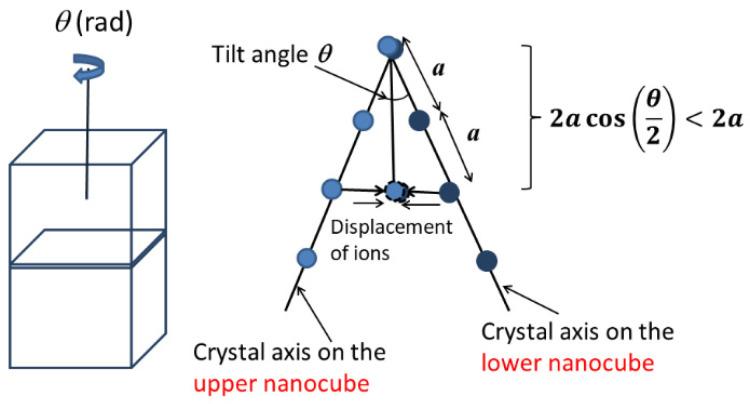
Misfit strain (**right**) caused by a small tilt angle *θ* between two attached BaTiO_3_ nanocubes (**left**). The right figure is the projection view of the interface from the above. Two corresponding crystal axes are shown in the figure. The small circles are Ba ions. Reprinted with permission from Ref. [86]. Copyright 2020, IOP Publishing.

In the self-assemblies of BaTiO_3_ nanocubes discussed in the previous section, strain may be introduced in each nanocube by the mechanism as shown in Figure 9 [86]. When the crystal axes of the neighboring nanocubes are titled by a small angle *θ*, the corresponding crystal axes would be shifted by a half angle *θ*/2 to align each other (Figure 9). Then, compressive strain appears for each crystal axis because the distance between neighboring ions becomes shorter. The compressive strain ($u_{m}$) is approximately expressed as follows [86].

(1) $$u_{m}=\cos\left(\frac{\theta }{2}\right)-1\;\leq 0$$

where negative value means the compressive strain. The tilt angle *θ* was experimentally estimated to be less than 10° [123]. Accordingly, $-0.0038\leq u_{m}\leq 0$. If $u_{m}=-0.002$ (θ=7.2°) is assumed, the magnitude of flexoelectric polarization (P) is estimated as

(2) $$P=\mu \frac{\partial \epsilon }{\partial x}\approx -\frac{\mu \cdot u_{m}}{\delta }\approx 2.67\;(C\;{m^{-}}^{2})\;$$

where μ is the flexoelectric coefficient (μ≈10 μC m^−1^ for BaTiO_3_ at room temperature [132]), $\frac{\partial \epsilon }{\partial x}$ is the strain gradient, and δ is the width of the strain region (δ≈d/2 is assumed, where d is the size of a nanocube (d=15 nm)) [86,87]. The estimated magnitude of flexoelectric polarization is about one order of magnitude larger than the spontaneous polarization of BaTiO_3_ [127]. Thus, it is expected that dielectric response of a BaTiO_3_ nanocube in the self-assemblies is mainly determined by that of the flexo-electric polarization when the flexoelectric polarization is perpendicular to the applied alternating electric field [87]. The flexoelectric polarization exists near each surface of a nanocube, and a total of six vectors of the flexoelectric polarizations exist inside a nanocube because there are six interfaces. For the flexoelectric polarization parallel to the applied alternating electric field, there is no contribution to the dielectric constant because there is a mismatch of strain at the interface, as the changes of strain at the interface is different between the two attaching nanocubes [87]. In this case, ferroelectric polarization of a BaTiO_3_ nanocube contributes to the dielectric constant. Four vectors of the flexoelectric polarization are perpendicular to the applied alternating electric field, and two vectors are parallel to the applied electric field. Accordingly, the dielectric constant (ε) is estimated as follows [87].

(3) $$\varepsilon \approx \frac{2}{3}\varepsilon_{flexo}+\frac{1}{3}\varepsilon_{ferro}$$

where $\varepsilon_{flexo}$ is the dielectric constant due to the flexoelectric polarization perpendicular to the applied electric field, and $\varepsilon_{ferro}$ is the dielectric constant due to the ferro-electric polarization parallel to the applied electric field.

**Figure 10:**
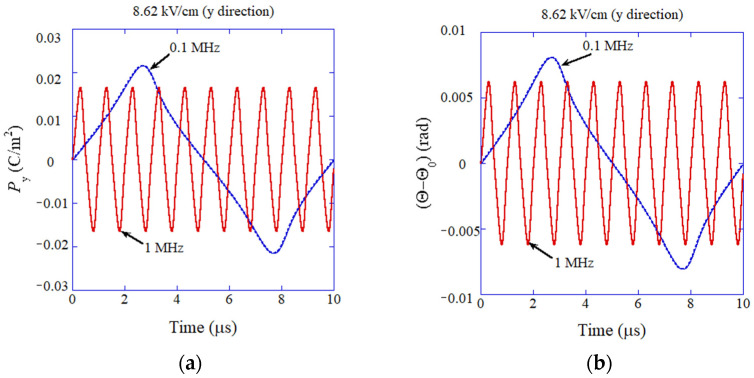
The results of numerical simulation of the ODE model for dynamic response of flexoelectric polarization of BaTiO_3_ nanocubes (**a**) The component of polarization parallel to the applied electric field (y-direction). (**b**) The angle (Θ) of polarization. Reprinted with permission from Ref. [87]. Copyright 2022, MDPI.

In order to calculate dielectric constant as a function of frequency of applied electric field to compare with the experimental data, an ODE model for dielectric response of flexoelectric polarization initially perpendicular to the applied alternating electric field is constructed [87]. The ODE model is simply the equation of rotational motion for the electric dipole. The following torques on an electric dipole are considered: the torque due to applied alternating electric field, the restoring torque due to anharmonic potential, and the damping torque [86,87]. As the anharmonic potential, the harmonic potential plus the nonlinear Lorentzian attractive-potential is assumed [87]. The mathematical model is briefly described in Section 7.3. Examples of the results of the numerical simulations are shown in Figure 10 [87]. Temporal variation of polarization as well as the angle of polarization relative to the direction perpendicular to the applied alternating electric field are easily simulated numerically using the ODE model, while it is computationally much more difficult using a PDE model [134,135,136,137,138,139]. A PDE model is also briefly described in Section 7.4. From Figure 10, the amplitude of variation of polarization is larger for lower frequency. It is due to the nonlinear potential and less damping caused by smaller angular velocity [87]. Accordingly, dielectric constant is larger for lower frequency. It is also seen in Figure 10 that the waveforms deviate significantly from the sinusoidal function, especially for the lower frequency due to the nonlinear potential.

**Figure 11:**
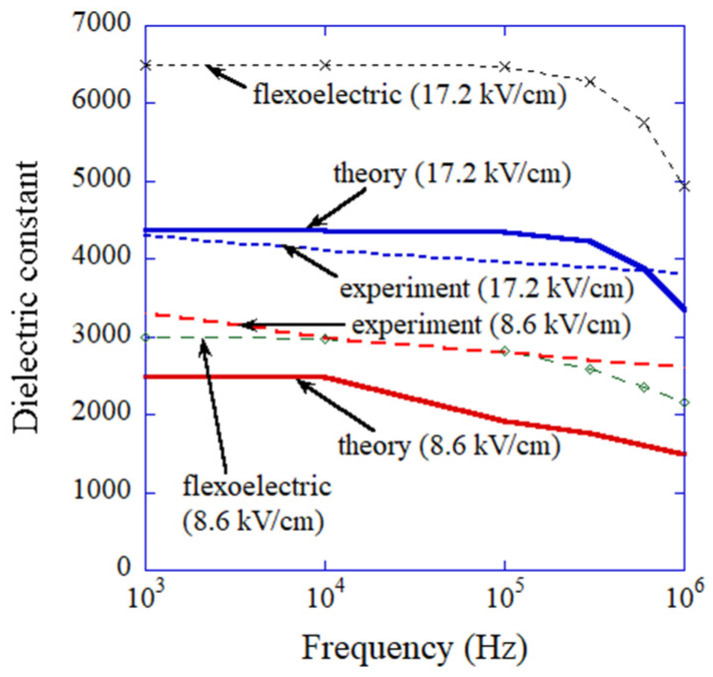
The dielectric constant according to the numerical simulations of the ODE model for dynamic response of flexoelectric polarization of BaTiO_3_ nanocubes taking into account the presence of ferroelectric polarization the results solely by flexoelectric polarization are also shown. The experimental data are also shown for comparison. Reprinted with permission from Ref. [87]. Copyright 2022, MDPI.

The dielectric constant due to flexoelectric polarization ($\varepsilon_{flexo}$) is calculated as follows.

(4) $$\varepsilon_{flexo}\approx \frac{{\left(P_{y}\right)}_{amp}}{E_{0}}$$

where ${\left(P_{y}\right)}_{amp}$ is the amplitude of temporal variation of $P_{y}$ which is the component of polarization parallel to the applied electric field, and $E_{0}$ is the amplitude of the applied alternating electric field. With regard to $\varepsilon_{ferro}$ in Equation (3), it is assumed as $\varepsilon_{ferro}\approx 1500$ according to the numerical calculations in Reference [140] and the flat frequency dependence of the dielectric constant for BaTiO_3_ ceramics without domain contribution [141,142]. The results of the numerical simulations on dielectric constant as a function of frequency of the applied alternating electric field is shown in Figure 11 with the experimental data [87,122]. The results of the numerical simulations nearly agree with the experimental data. This suggests that the ODE model is consistent. However, the nonlinear potential used in the ODE model was determined to fit the experimental data. Thus, to validate the ODE model, comparison with the results of numerical simulations based on more rigorous PDE models is strongly required. Furthermore, the coexistence of flexo-and ferro-electric polarizations should be confirmed experimentally. These are the demerits of an ODE model, as an ODE model is not fully based on the first principles.

## 5. Ultrasound-Assisted Sintering

Cold sintering has been a popular topic since Randall and his research group at Pennsylvania State University reported it in 2016 [143,144,145,146,147,148]. Cold sintering is densification of ceramic particles under high pressure and limited temperature with the help of liquid water such as in the geological formation of sedimentary rocks [145]. Ultrasound-assisted sintering has a possibility to further improve the cold sintering process [149,150,151]. However, the detailed mechanism for ultrasound-assisted sintering is still under debate. It has been known that ultrasonic irradiation results in softening of solid materials called acoustic softening [152,153]. In acoustic softening, it has been suggested that accelerated dislocation motion by ultrasonic irradiation plays an important role [153]. Furthermore, the increase in dislocation density by ultrasonic irradiation has been experimentally reported [154,155]. Thus, in ultrasound-assisted sintering, it is expected that dislocations may play some role. In order to study the mechanism of ultrasound-assisted sintering, an ODE model is constructed [88]. Numerical simulations were performed under the experimental condition of ultrasound-assisted sintering of silver nanoparticles by Wang et al. [149]. In the experiment, silver nanoparticles of about 20 nm in diameter were irradiated by ultrasound of 40 kHz with the vibration amplitudes of 3, 6, 9, and 12 μm for 7 min under static pressure of 10 MPa at 120 °C. The resulting porosity of the sample was about 15%, 8%, and 4.5% for the vibration amplitude of 0 (without ultrasound), 6, and 12 μm, respectively [149].

The ODE model of ultrasound-assisted sintering is developed from the model of solid-state sintering of Kraft and Riedel [156], coupled with the model of evolutions of mobile and immobile dislocations as well as that of vacancies of Buzolin et al. [157] and Lindgren et al. [158]. The outline of the ODE model is as follows [88]. The densification rate (sintering rate) is given by the relative density of the sample and the plastic strain rate. The plastic strain rate is given by the static stress, stress produced by the ultrasonic wave, sintering stress, and bulk viscosity of the sample. The bulk viscosity is a nonlinear function of the effective stress and depends on dislocation density as well as concentration of vacancies [88]. The grain growth rate is given by the grain radius, specific energy of the grain boundary, and grain boundary mobility. The evolution of the mobile dislocation density is given by the production rate, rate of immobilization, and rate of annihilation. The evolution of immobile dislocation density is given by the rate of immobilization of mobile dislocations, and rate of annihilation.

**Figure 12:**
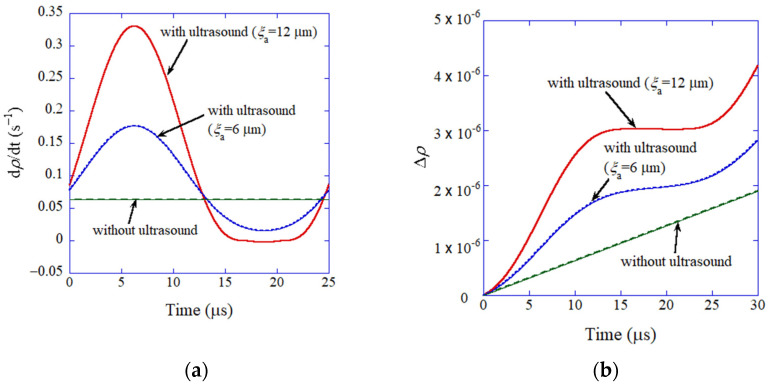
The results of numerical simulations of the ODE model for ultrasound-assisted sintering of silver nanoparticles as a function of time (**a**) The densification rate. (**b**) The change in the relative density. Reprinted with permission from Ref. [88]. Copyright 2021, AIP Publishing.

The results of the numerical simulations have indicated that total dislocation density does not increase when the grain size is smaller than about 10 μm because mobile dislocations are immediately immobilized [88]. Thus, under the experimental conditions of Wang et al. [149], there is no increase in total dislocation density, as the initial grain size is as small as 20 nm. In other words, the role of dislocations on ultrasound-assisted sintering would be minor. In contrast, the increase in relative density is enhanced by ultrasonic irradiation because of the nonlinear bulk viscosity as a function of effective stress (Figure 12) [88]. The main effect of ultrasonic irradiation is, however, the decrease in pore size at the initial stage of ultrasonic irradiation, which results in higher final relative density (lower final porosity). The ODE model is useful to discuss the mechanism of ultrasound-assisted sintering. However, the ODE model needs to be validated through comparison with the experimental data or results of the first-principles calculations.

**Figure 13:**
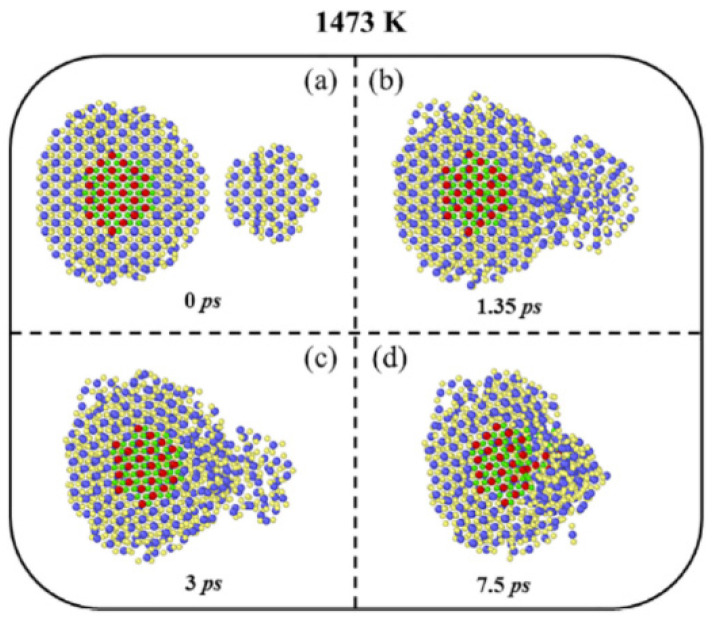
Snapshots from the results of molecular dynamics simulations of the sintering process for TiO_2_ nanoparticles. Reprinted with permission from Ref. [159]. Copyright 2022, Elsevier.

Next, molecular dynamics simulations of the sintering process of TiO_2_ nanoparticles in the absence of ultrasound are briefly discussed (Figure 13) [159]. Details of the sintering process of two nanoparticles are captured by the molecular dynamics simulations. Conversely, the macroscopic quantities such as relative density are hardly calculated by the molecular dynamics simulations. Furthermore, molecular dynamics simulations computationally cost much more compared to simulations of an ODE model.

## 6. Dynamics of a Gas Parcel in a Thermoacoustic Engine

At the end of the 20th century, there were two popular topics in acoustics. One is SBSL discussed in Section 2, and the other is thermoacoustic engines discussed in this section. Waste heat at relatively low temperatures in factories is still disposed of without reuse. One of the candidates to reuse such waste heat is a thermoacoustic engine which converts heat into sound [160]. The generated sound can be used to cool the environment by using the (inverse) thermoacoustic effect. A typical thermoacoustic engine consists of a looped pipe (traveling-wave type) or a straight pipe (standing-wave type) in which a stack is mounted [161]. A stack consists of many narrow tubes parallel to the length direction. When the temperature difference between the two sides of a stack is above the critical value, sound is generated. When another stack is mounted in the pipe at an appropriate position, the generated sound makes a temperature difference between the two sides of the stack. In other words, one side could be cooled considerably, which can be used as a cooler [162]. It is also possible to obtain electric power from the generated sound using a linear alternator [163].

**Figure 14:**
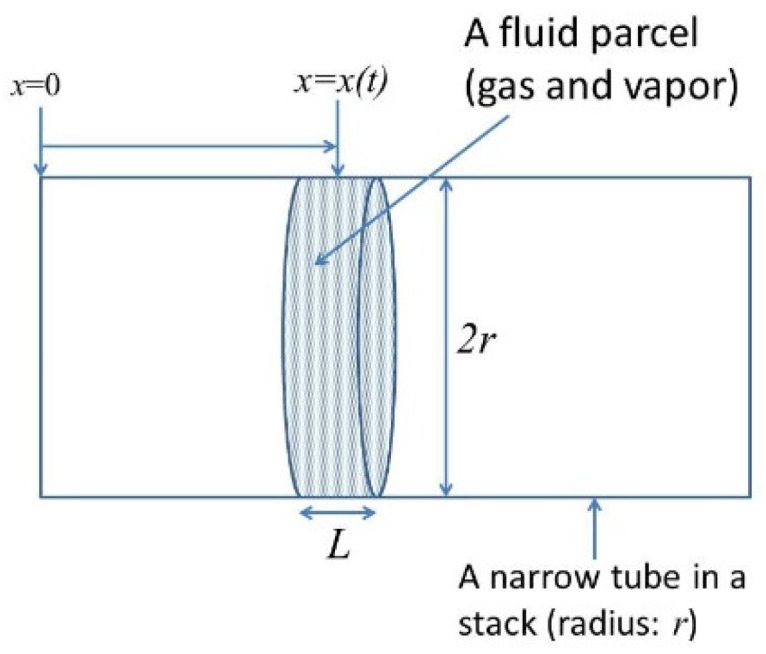
A fluid (a mixture of gas and water vapor) parcel in a narrow tube in a stack of a thermoacoustic engine. Reprinted with permission from Ref. [90]. Copyright 2017, the Acoustical Society of America.

It has been reported that the critical temperature difference to generate sound considerably decreases by using a stack wetted with water [164,165,166]. However, the mechanism is still under debate. In order to study the mechanism, an ODE model is constructed as follows [90,91]. In the model, expansion and contraction of a fluid parcel as well as its translational motion are numerically simulated inside a narrow tube of a stack (Figure 14) [90]. A fluid parcel consists of permanent gas such as air and water vapor (if present). In the calculation of instantaneous velocity of a fluid parcel, a gradient of velocity amplitude is used, which is calculated by the Rott equations [90,91]. In the calculation of instantaneous pressure inside a fluid parcel, the gradient of pressure amplitude is used, which is also calculated by the Rott equation. The instantaneous temperature inside a fluid parcel is calculated by the instantaneous thermal energy of a fluid parcel, which is calculated by pV work done by the surrounding fluid, energy change due to thermal conduction between the fluid parcel and the wall of a narrow tube, and energy change due to evaporation or condensation of water vapor at the wall for a wet stack [90,91]. The number of water vapor molecules inside a fluid parcel changes by evaporation or condensation, which also influences the instantaneous temperature of a fluid parcel.

**Figure 15:**
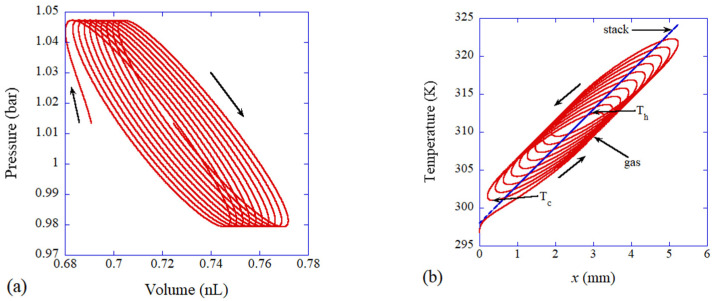
The results of the numerical simulation of the ODE model for a gas parcel in a traveling-wave thermoacoustic engine near the cold end of a dry stack (**a**) p-V diagram. (**b**) T-x diagram. Reprinted with permission from Ref. [91]. Copyright 2021, AIP Publishing.

**Figure 16:**
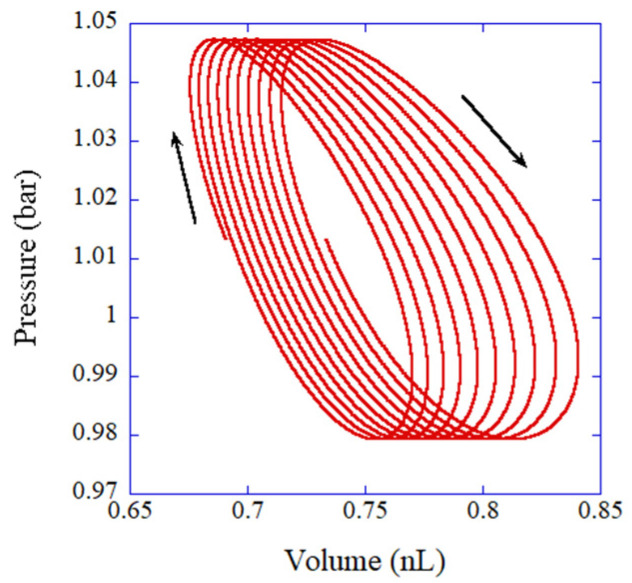
The p-V diagram according to the numerical simulation of the ODE model for a fluid parcel in a traveling-wave thermoacoustic engine near the cold end of a wet stack. Reprinted with permission from Ref. [91] Copyright 2021, AIP Publishing.

Examples of the results of the numerical simulations of the ODE model are shown in Figure 15 and Figure 16 for a dry and wet stack, respectively [91]. For both dry and wet stacks, the trajectories in p-V diagram move in a clockwise manner, which means that net pV work done by a fluid parcel to the surroundings is positive. In other words, a fluid parcel radiates sound wave into the surroundings, which is the mechanism for a thermoacoustic engine. The area surrounded by a trajectory in the p-V diagram in one acoustic cycle, which equals the net pV work, is larger in a wet stack compared to that in a dry stack (Figure 15 and Figure 16). In other words, acoustic energy radiated from a fluid parcel is larger in a wet stack than that in a dry stack. The reason for the larger acoustic energy in a wet stack is the lager amplitude of volume oscillation of a fluid parcel because water vapor evaporates during the expansion of a fluid parcel and condenses during the compression of a fluid parcel in a travelling thermoacoustic engine [90]. The mechanism is easily clarified, which is the merits of an ODE model. Using a more rigorous PDE model [167,168], it is much more difficult to clarify the dynamic mechanism. Conversely, the ODE model needs to be validated through comparison with the experimental data or results of first-principles calculations because the ODE model contains an unknown parameter [90,91].

From T-x diagram in Figure 15b, it is seen that the thermodynamic cycle in a traveling thermoacoustic-engine deviates significantly from the ideal Stirling cycle in which isothermal processes are present [161]. From the ODE model, these dynamic features can be easily clarified.

## 7. Mathematical Models

### 7.1. A Cavitation Bubble (ODE Model) [95]

For the temporal variation of bubble radius (R), the Keller equation (Equation (5)) is employed.

(5) $$\left(1-\frac{\dot{R}}{c_{\infty }}\right)R\ddot{R}+\frac{3}{2}{\dot{R}}^{2}\left(1-\frac{\dot{R}}{3c_{\infty }}\right)=\frac{1}{\rho_{L,\infty }}\left(1+\frac{\dot{R}}{c_{\infty }}\right)\left[p_{B}-p_{s}\left(t\right)-p_{\infty }\right]+\frac{R}{c_{\infty }\rho_{L,\infty }}\frac{dp_{B}}{dt}$$

where “dot” denotes the time derivative (*d*/*dt*), $c_{\infty }$ is the sound velocity in liquid at ambient condition, $\rho_{L,\infty }$ is liquid density at ambient condition, $p_{B}$ is liquid pressure at the bubble wall, $p_{s}\left(t\right)$ is instantaneous acoustic pressure, and $p_{\infty }$ is the ambient pressure. $p_{B}$ is calculated as follows.

(6) $$p_{B}=p_{g}+p_{v}-\frac{2\sigma }{R}-\frac{4\mu_{L}\dot{R}}{R}$$

where $p_{g}$ and $p_{v}$ are partial pressures of non-condensable gas and vapor, respectively, σ is surface tension, and $\mu_{L}$ is the liquid viscosity. The pressure inside a bubble ($p_{in}=p_{g}+p_{v}$) is calculated by the van der Waals equation of state (Equation (7)).

(7) $$\left[p_{in}+\frac{a_{v}}{v^{2}}\right]\left(v-b_{v}\right)=R_{g}T$$

where $a_{v}$ and $b_{v}$ are the van der Waals constants, v is the molar volume, $R_{g}$ is the gas constant, and T is the temperature inside a bubble. The molar volume v is calculated as follows.

(8) $$v=\frac{4\pi R^{3}}{3}\cdot \frac{N_{A}}{n_{t}}$$

where $N_{A}$ is the Avogadro number, and $n_{t}$ is the total number of molecules inside a bubble. The temperature (T) inside a bubble is approximately calculated from internal thermal energy (E) of a bubble by the following equation.

(9) $$E=\frac{T}{N_{A}}\sum_{\alpha }n_{\alpha }C_{V,\alpha }-{\left(\frac{n_{t}}{N_{A}}\right)}^{2}\frac{a_{v}}{V}$$

where $n_{\alpha }$ is the number of molecules of species α inside a bubble, $C_{V,\alpha }$ is the molar heat capacity at constant volume of species α, the summation is for all the gas and vapor species inside a bubble, and V is the bubble volume.

The change in the internal thermal energy (ΔE) of a bubble is given as follows.

(10) $$\Delta E=-p_{in}\Delta V+4\pi R^{2}\dot{m}e_{H_{2}O}\Delta t+4\pi R^{2}\kappa {\left.\frac{\partial T}{\partial r}\right|}_{r=R}\Delta t+\frac{4}{3}\pi R^{3}\Delta t\underset{\gamma }{\sum }\left(r_{\gamma b}-r_{\gamma f}\right)\Delta H_{\gamma f}+\underset{\overset{´}{\alpha }}{\sum }e_{\overset{´}{\alpha }}\Delta n_{\overset{´}{\alpha }}+\left[-\frac{3}{5}M_{in}\dot{R}\ddot{R}\right]\Delta t$$

where ΔV is the change in the bubble volume, $\dot{m}$ is the rate of non-equilibrium evaporation at the bubble wall, $e_{H_{2}O}$ is the energy carried by an evaporating or condensing vapor molecule, Δt is the time step in the numerical integration, κ is the thermal conductivity of a mixture of gases and vapor, ${\left.\frac{\partial T}{\partial r}\right|}_{r=R}$ is the temperature gradient inside a bubble at the bubble wall, $r_{\gamma b}$ and $r_{\gamma f}$ are the backward and forward reaction rates, respectively, of chemical reaction γ per unit volume and unit time, $\Delta H_{\gamma f}$ is the enthalpy change in the forward chemical reaction, $e_{\overset{´}{\alpha }}$ is the energy carried by a diffusing gas molecule of the species α, $\Delta n_{\overset{´}{\alpha }}$ is the number of molecules of species α diffusing into a bubble in time Δt, and $M_{in}$ is the total mass of gases and vapor inside a bubble. The first term on the right side of Equation (10) is the pV work done by the surrounding liquid on a bubble. The second term is the energy carried by evaporating vapor molecules into a bubble. The third term is the energy change due to thermal conduction. The fourth term is the heat of chemical reactions. The fifth term is the energy carried by diffusing gas molecules. The last term is included only when the quantity in the brackets is positive and is heating due to the decrease in kinetic energy of gases and vapor inside a collapsing bubble. For more details, see Reference [95].

The ODE model is numerically solved simply by the Euler method as follows.

(11) $$R\left(t+\Delta t\right)=R\left(t\right)+\dot{R}\left(t\right)\Delta t\;$$

(12) $$\dot{R}\left(t+\Delta t\right)=\dot{R}\left(t\right)+\ddot{R}\left(t\right)\Delta t\;$$

where $\ddot{R}\left(t\right)$ is given by Equation (5).

### 7.2. A Cavitation Bubble (PDE Model) [109,169]

For the temporal variation of bubble radius (R), the Rayleigh–Plesset equation (Equation (13)) is employed.

(13) $$R\ddot{R}+\frac{3}{2}{\dot{R}}^{2}=\frac{1}{\rho_{L,\infty }}\left[p_{B}-p_{s}\left(t\right)-p_{\infty }\right]+\frac{R}{c_{\infty }\rho_{L,\infty }}\frac{dp_{B}}{dt}$$

For the interior of a bubble, spatial variations of temperature, pressure, and density are numerically calculated by solving the PDEs of fluid mechanics of two kinds of gas components, the inert gas and the water vapor, as follows.

(14) $$\frac{\partial \rho_{1}}{\partial t}+\frac{1}{r^{2}}\frac{\partial }{\partial r}\left(r^{2}\rho_{1}v_{1}\right)=0$$

(15) $$\frac{\partial \rho }{\partial t}+\frac{1}{r^{2}}\frac{\partial }{\partial r}\left(r^{2}\rho v\right)$$

(16) $$\frac{\partial \left(\rho v\right)}{\partial t}+\frac{1}{r^{2}}\frac{\partial }{\partial r}\left(r^{2}\rho v\right)+\frac{\partial p}{\partial r}=\frac{1}{r^{2}}\frac{\partial }{\partial r}\left(r^{2}\tau_{rr}\right)+\frac{\tau_{rr}}{r}$$

(17) $$\frac{\partial E}{\partial t}+\frac{1}{r^{2}}\frac{\partial }{\partial r}\left\{r^{2}\left[E+p\right]v+q\right\}=\frac{1}{r^{2}}\frac{\partial }{\partial r}\left(r^{2}v\tau_{rr}\right)$$

where $\rho_{1}$ and $\rho_{2}$ are density of vapor and inert gas, respectively, r is the radial coordinate, $v_{1}$ and $v_{2}$ are radial velocity of vapor and inert gas, respectively, $\rho =\rho_{1}+\rho_{2}$, v is the average velocity ($\rho v=\rho_{1}v_{1}+\rho_{2}v_{2}$), p is pressure, $\tau_{rr}=\left(4\mu /3\right)\left(\frac{\partial v}{\partial r}-v/r\right)$, μ is viscosity, E is the total energy density, and q is the heat flux. Equations (14) and (15) are the equation of continuity of vapor and that of the mixture of gas and vapor, respectively. Equation (16) is the equation of motion. Equation (17) is the energy equation. In the PDE model, energy equation in liquid is also solved numerically. The above PDEs are numerically solved using fixed elements. For more details, please see References [109,169].

### 7.3. Flexoelectric Polarization (ODE Model) [87]

Dynamic response of flexoelectric polarization is simply modeled by the following equation of rotational motion for the electric dipole.

(18) $$I\frac{d^{2}\theta }{dt^{2}}=\left(p\cos\theta \right)E_{0}\sin\left(\omega_{E}t\right)-k\left(\theta -\theta_{0}\right)-\frac{\beta \left(\theta -\theta_{0}\right)}{{\left[1+\zeta {\left(\theta -\theta_{0}\right)}^{2}\right]}^{2}}-\lambda \frac{d\theta }{dt}$$

where I is the (virtual) moment of inertia, θ is the angle of polarization relative to *x*-axis, t is time, p is (virtual) electric dipole moment, which is related to flexoelectric polarization (P) as p=PV, V is volume, $E_{0}$ is the amplitude of the applied alternating electric field, $\omega_{E}$ is the angular frequency of applied electric field, k is the spring constant for angular harmonic potential, $\theta_{0}$ is the equilibrium angle of polarization, β and ζ are coefficients for angular Lorentzian potential, and λ is the angular damping constant. The component of polarization parallel to the applied electric field (*y* direction) is $P_{y}=\left|P\right|\sin\theta$. The ODE can be numerically solved by the Euler method as well as by a more sophisticated method such as the Runge–Kutta method.

### 7.4. Flexoelectric Polarization (PDE Model) [136]

The following full equations of motion are derived from the time-dependent Ginzburg–Landau equations.

(19) $$-\frac{1}{\Gamma }\frac{\partial P_{x}}{\partial t}=2\alpha_{1}P_{x}+4\alpha_{11}P_{x}^{3}+2\alpha_{12}P_{x}P_{y}^{2}+6\alpha_{111}P_{x}^{5}+\alpha_{112}\left(4P_{x}^{3}P_{y}^{2}+2P_{x}P_{y}^{4}\right)\;\;-2P_{x}\left(Q_{11}\sigma_{xx}^{e}+Q_{12}\sigma_{yy}^{e}\right)-Q_{44}\sigma_{xy}^{e}P_{y}-f_{11}\left(\frac{\partial u_{xx}}{\partial x}\right)-f_{12}\left(\frac{\partial u_{yy}}{\partial x}\right)\;\;-f_{44}\left(\frac{\partial u_{xy}}{\partial y}\right)-\lambda_{1}\nabla^{2}P_{x}+\lambda_{2}\nabla^{2}\left(\nabla^{2}P_{x}\right)+\frac{\partial \varphi }{\partial x}$$

(20) $$-\frac{1}{\Gamma }\frac{\partial P_{y}}{\partial t}=2\alpha_{1}P_{y}+4\alpha_{11}P_{y}^{3}+2\alpha_{12}P_{y}P_{x}^{2}+6\alpha_{111}P_{y}^{5}+\alpha_{112}\left(4P_{y}^{3}P_{x}^{2}+2P_{y}P_{x}^{4}\right)\;\;-2P_{y}\left(Q_{11}\sigma_{yy}^{e}+Q_{12}\sigma_{xx}^{e}\right)-Q_{44}\sigma_{xy}^{e}P_{x}-f_{11}\left(\frac{\partial u_{yy}}{\partial y}\right)\;\;-f_{12}\left(\frac{\partial u_{xx}}{\partial y}\right)-f_{44}\left(\frac{\partial u_{xy}}{\partial x}\right)-\lambda_{1}\nabla^{2}P_{y}+\lambda_{2}\nabla^{2}\left(\nabla^{2}P_{y}\right)+\frac{\partial \varphi }{\partial y}$$

where Γ is a kinetic coefficient, $\alpha_{1},\;\alpha_{11},\;\alpha_{12},\;\alpha_{111},\;\alpha_{112},\;\lambda_{1},\lambda_{2}$ are coefficients for the Landau expansion, $Q_{ij}$ are the electrostrictive constants that couple strains and squares of polarizations, $\sigma_{ij}^{e}$ represents the electrostrictive part of the stress tensor, $u_{ij}$ represents the linearized strain components, $\nabla^{2}=\left(\partial^{2}/\partial x^{2}\right)+\left(\partial^{2}/\partial y^{2}\right)$ is the Laplacian operator, and φ is the electrostatic potential.

The displacement field dynamics are given by the dissipative force balance equations as follows.

(21) $$\rho \frac{\partial^{2}u_{x}}{\partial t^{2}}-\eta \nabla^{2}\frac{\partial u_{x}}{\partial t}=\frac{\partial \sigma_{xx}}{\partial x}+\frac{\partial \sigma_{xy}}{\partial y}$$

(22) $$\rho \frac{\partial^{2}u_{y}}{\partial t^{2}}-\eta \nabla^{2}\frac{\partial u_{y}}{\partial t}=\frac{\partial \sigma_{xy}}{\partial x}+\frac{\partial \sigma_{yy}}{\partial y}$$

where ρ is density, $u_{i}$ represents i component of displacement, η is a viscosity that is used to drive the system toward mechanical equilibrium $\frac{\partial \sigma_{ij}}{\partial x_{j}}=0$, and $\sigma_{ij}$ represents stress components.

For a system without free charge, Gauss’s law leads to the following constraint.

(23) $$\vec{\nabla }\cdot (-\varepsilon_{b}\vec{\nabla }\varphi +\vec{P})=0$$

$\varepsilon_{b}$ is background dielectric permittivity of the material.

In the PDE model, Equations (19)–(23) are used to study the dynamics of the polarization. To simulate the domain patterns, the PDEs are numerically solved using a finite difference method. For more details, please see Reference [136].

## 8. Conclusions

Numerical simulations with ODE modeling are sometimes superior to the first-principles calculations because the mechanisms are more easily clarified, and scanning the large parameter space is much easier. The reasons are that an ODE model is computationally more economical, and important factors are more easily traced. Conversely, an ODE model needs validation through comparison with the experimental data or results of first-principles calculations because an ODE model is not fully based on the first principles.

## Data Availability

Not applicable.
